# Interpretations of Environmental Microbial Community Studies Are Biased by the Selected 16S rRNA (Gene) Amplicon Sequencing Pipeline

**DOI:** 10.3389/fmicb.2020.550420

**Published:** 2020-10-23

**Authors:** Daniel Straub, Nia Blackwell, Adrian Langarica-Fuentes, Alexander Peltzer, Sven Nahnsen, Sara Kleindienst

**Affiliations:** ^1^Microbial Ecology, Center for Applied Geoscience, Department of Geosciences, University of Tübingen, Tübingen, Germany; ^2^Quantitative Biology Center (QBiC), University of Tübingen, Tübingen, Germany

**Keywords:** 16S rRNA, amplicon sequencing, environmental samples, bioinformatics, nf-core/ampliseq

## Abstract

One of the major methods to identify microbial community composition, to unravel microbial population dynamics, and to explore microbial diversity in environmental samples is high-throughput DNA- or RNA-based 16S rRNA (gene) amplicon sequencing in combination with bioinformatics analyses. However, focusing on environmental samples from contrasting habitats, it was not systematically evaluated (i) which analysis methods provide results that reflect reality most accurately, (ii) how the interpretations of microbial community studies are biased by different analysis methods and (iii) if the most optimal analysis workflow can be implemented in an easy-to-use pipeline. Here, we compared the performance of 16S rRNA (gene) amplicon sequencing analysis tools (i.e., Mothur, QIIME1, QIIME2, and MEGAN) using three mock datasets with known microbial community composition that differed in sequencing quality, species number and abundance distribution (i.e., even or uneven), and phylogenetic diversity (i.e., closely related or well-separated amplicon sequences). Our results showed that QIIME2 outcompeted all other investigated tools in sequence recovery (>10 times fewer false positives), taxonomic assignments (>22% better F-score) and diversity estimates (>5% better assessment), suggesting that this approach is able to reflect the *in situ* microbial community most accurately. Further analysis of 24 environmental datasets obtained from four contrasting terrestrial and freshwater sites revealed dramatic differences in the resulting microbial community composition for all pipelines at genus level. For instance, at the investigated river water sites *Sphaerotilus* was only reported when using QIIME1 (8% abundance) and *Agitococcus* with QIIME1 or QIIME2 (2 or 3% abundance, respectively), but both genera remained undetected when analyzed with Mothur or MEGAN. Since these abundant taxa probably have implications for important biogeochemical cycles (e.g., nitrate and sulfate reduction) at these sites, their detection and semi-quantitative enumeration is crucial for valid interpretations. A high-performance computing conformant workflow was constructed to allow FAIR (Findable, Accessible, Interoperable, and Re-usable) 16S rRNA (gene) amplicon sequence analysis starting from raw sequence files, using the most optimal methods identified in our study. Our presented workflow should be considered for future studies, thereby facilitating the analysis of high-throughput 16S rRNA (gene) sequencing data substantially, while maximizing reliability and confidence in microbial community data analysis.

## Introduction

The ribosomal 16S rRNA gene is a phylogenetic marker that has been analyzed extensively within the last decade due to its presence in all microorganisms (Hugenholtz et al., 1998), and due to a combination of variable regions, influenced by the evolutionary clock that allow differentiation of taxa, with conserved regions, for universal priming (Head et al., 1998). Since the dawn of next-generation sequencing methods, the cost of nucleotide sequencing has decreased dramatically (Wetterstrand, 2018) and DNA- or RNA-based 16S rRNA (gene) amplicon sequencing is becoming more and more affordable. Initially, 454 pyrosequencing was employed but, after resolving early limitations, relatively short Illumina sequencing is currently dominating (Claesson et al., 2010; D’Amore et al., 2016) because of higher sequence quality and cost advantages.

16S rRNA (gene) amplicon sequencing analysis pipelines are required to be user-friendly and to provide the best output possible. Criteria for optimal results include the recovery of all 16S rRNA (gene) amplicon sequences and taxa (full sensitivity) with no false positive detection (full specificity). Also, *in situ* relative abundances are ideally perfectly represented. However, all current analysis methods suffer from imperfect recall (not all sequences or taxa are detected) or imperfect precision (additional false sequences or taxa are detected) (Callahan et al., 2016) that originate from a diverse set of frequent shortcomings of the entire workflow. These include biases in sample preparation (e.g., DNA extraction, PCR, sequencing library preparation), suboptimal experimental design (e.g., amplicon and primer selection), erroneous sequences produced by the sequencing method and the bioinformatics analysis strategy (Kozich et al., 2013; Wesolowska-Andersen et al., 2014; de Muinck et al., 2017; Laursen et al., 2017; Almeida et al., 2018; Nearing et al., 2018).

The scientific literature suggesting software applications for the analysis of 16S rRNA (gene) sequencing data is continuously growing and many methods have been proposed. Three major types of methods can be distinguished: (i) Clustering of sequencing reads to obtain operational taxonomic units (OTUs), (ii) generation of amplicon sequence variants (ASVs) using error-corrected reads, or (iii) direct taxonomic classification of raw reads. Tools such as UPARSE (Edgar, 2013), Swarm (Mahé et al., 2015) or VSEARCH (Rognes et al., 2016) cluster sequences with a given similarity (e.g., ≥97%) into OTUs and are also integrated in overarching frameworks such as Quantitative Insights Into Microbial Ecology (QIIME) (Caporaso et al., 2010b), Mothur (Schloss et al., 2009), USEARCH (Edgar, 2010) or FROGS (Escudié et al., 2017). Clustering sequences masks biological variation and was shown to produce artifacts, e.g., QIIME reportedly produced inflated numbers of OTUs with standard parameters (Edgar, 2017). More recently developed methods such as UNOISE (Edgar and Flyvbjerg, 2015), Divisive Amplicon Denoising Algorithm (DADA2) (Callahan et al., 2016), or Deblur (Amir et al., 2017) compute ASVs that are generally considered to be a more detailed view of OTUs as produced by QIIME1 or Mothur (Callahan et al., 2017). Ideally, ASVs represent actual amplicon sequences with single-nucleotide resolution that originate from each 16S rRNA gene copy of each species so that one species might be represented by several ASVs (Větrovský and Baldrian, 2013). Still, 16S rRNA genes that do not differ in their amplified sequence cannot be resolved. DADA2 and Deblur are available in QIIME2 (Bolyen et al., 2019), the successor of QIIME (called from here on QIIME1). Yet another group of tools directly classifies sequencing reads into taxonomic bins without OTU/ASV generation. These tools are typically used for shotgun metagenomics but sometimes also for amplicon analysis, e.g., MEGAN (Mitra et al., 2011), Kraken2 (Lu and Salzberg, 2020) or Centrifuge (Cuscó et al., 2018; Khachatryan et al., 2020). Recovering representative sequences such as OTUs or ASVs allows for further analysis like constructing phylogenetic trees or performing targeted analyses, such as searching for the same sequence or related sequences in other data sets, that is not possible when using direct read classification. However, direct taxonomic classification can be straight forward with functional gene sequences that do not have elaborate reference databases like the 16S rRNA gene (DeSantis et al., 2006; Quast et al., 2013), e.g., for methane monooxygenase genes (*pmoA*, *mmoX*) or methanol dehydrogenase genes (*xoxF4*, *xoxF5*, *mxaF*) (Taubert et al., 2019).

Various bioinformatics tools complicate the selection and the choice of the analysis approach. Several studies comparing different 16S rRNA (gene) amplicon sequencing analysis methods and pipelines have been published over the years, many of those by authors of new tools who had to benchmark it against existing work (e.g., Edgar, 2013; Callahan et al., 2016; Escudié et al., 2017). Independent studies compared for example amplicon sequencing to shotgun metagenomics (Tessler et al., 2017), OTU clustering methods (Kopylova et al., 2016), OTU clustering to raw read classification (Siegwald et al., 2017), or OTU clustering to error-correcting ASV methods (Nearing et al., 2018; Prodan et al., 2020). Interestingly, also a meta-analysis of four published evaluations of metagenome and amplicon analysis software was published (Gardner et al., 2019). Some studies are only based on simplified mock communities and include no diverse samples (e.g., Gardner et al., 2019; Khachatryan et al., 2020); other studies test without mock datasets and therefore cannot compare the results to the underlying truth but focus on differences caused by the analysis methods (e.g., Sinclair et al., 2015; Tessler et al., 2017). The combination of independent analysis of mock and environmental samples from contrasting habitats as well as comprehensive comparisons at multiple levels (OTU/ASV, taxonomy, alpha- and beta diversity) is rare. Additionally, some of these studies are not addressed to microbiologists but rather to bioinformaticians or do not include detailed guidelines. Focusing on environmental samples from contrasting habitats, it was previously not systematically evaluated (i) what data analysis tools report the *in situ* microbial community composition most accurately, (ii) how the interpretations of microbial community studies differ among tools and (iii) whether an easy-to-use pipeline allowing reproducible and reliable results can be constructed.

In this study, we therefore aimed at identifying most suitable bioinformatics methods to examine microbial communities based on 16S rRNA (gene) amplicon sequencing data, while revealing differences caused by these methods with a focus on taxonomic identification and microbial diversity analysis. We subsequently aimed at implementing our findings into an open-access pipeline. Key elements for our comparisons included the accuracy of recovered 16S rRNA gene amplicon sequences, their taxonomic classification and their relative abundances. All these elements are essential for exploring microbial communities, predicting ecological relevance, identifying microbial key players involved in biochemical cycles or drawing conclusions about differences between communities. Here, we compared common tools that produce OTUs or ASVs, or directly classify reads taxonomically. Since a comparison of all existing tools is not realistic, one of our most important criteria for our tool selection was the adherence to the open source and permissive licensing models. We consider this important for the implementation of FAIR (Findable, Accessible, Interoperable, and Re-usable) (Wilkinson et al., 2016) and reproducible processing pipelines. While USEARCH (with UPARSE and UNOISE) is widespread, it is not open source software and has furthermore limitations for the software redistribution. Therefore, we chose OTU-clustering Mothur and QIIME1, ASV-producing QIIME2 (with DADA2 or Deblur) and MEGAN that was the first tool among read classifiers that was adopted to analyze amplicon data. We compared these tools with three mock datasets and 24 environmental samples. Additionally, we implemented our findings as an nf-core workflow (Ewels et al., 2019; Straub and Peltzer, 2019) to allow for execution in highly parallelized computing infrastructures, such as high-performance computing environments or compute clouds. Nf-core workflows strictly follow the FAIR principle (Wilkinson et al., 2016), come with high quality standards, and are fully based on open source software (Ewels et al., 2019).

## Materials and Methods

### Mock Test Datasets

To compare the performance of Mothur, QIIME1, QIIME2, and MEGAN, three mock datasets differing in microbial community composition, abundance distribution, and data quality were selected. All three datasets investigated the V4 region of the 16S rRNA gene and were sequenced by Illumina MiSeq. The “Balanced” dataset consisted of 57 bacteria and archaea from a broad range of habitats at even amounts of purified genomic DNA (Schirmer et al., 2015). The “Extreme” community contained 27 human gastrointestinal tract bacterial isolates at frequencies spanning six orders of magnitude and differing by as little as one nucleotide, for which 16S rRNA gene amplicons of individual cultures were quantified and pooled (Callahan et al., 2016). The “HMP” (Human Microbiome Project) dataset contained 21 well-separated strains in the human body with equimolar concentrations of 16S rRNA gene copies per genome (Kozich et al., 2013); the sequence quality was the lowest of the three mock datasets.

The Balanced dataset was retrieved from the European Nucleotide Archive (ENA), study PRJEB6244 sample M35 (SAMEA3298272) (Schirmer et al., 2015), the Extreme dataset SRR2990088 (Callahan et al., 2016) was downloaded from the Sequence Read Archive (SRA), and the HMP dataset, alias Mock1, was downloaded at mothur.org in set “130403” (Kozich et al., 2013). For the Balanced and HMP datasets, primers were removed from raw sequences and untrimmed sequences were discarded with Cutadapt v1.14 (Parada et al., 2016) wrapped by Trim Galore! v0.4.5 (Felix Krueger)^1^.

For the Extreme and HMP datasets, reference sequences were obtained from the Supplementary File “Supplementary Software” from Callahan et al. (2016). For the Balanced dataset, reference sequences for each species were retrieved from the “ribosomal RNA operon copy number database” (rrnDB) v5.4 (Stoddard et al., 2015). For QIIME2 in combination with Deblur, all recovered amplicon sequences had equal lengths determined by an input parameter and, therefore, reference sequences were truncated to the same length.

Further details on mock datasets and trimmed primers can be found in the Supplementary Table 1.

### Sample Collection and DNA Extraction

Groundwater, soil, river sediment, and river water were each sampled in triplicate at two sites.

#### Groundwater

Groundwater was collected from the monitoring well in Haslach (sampling site 1) using a submersible pump operating at the top of the screened section in 70 m depth below the well head (47.7 m below the water table) with a flow rate of about 0.1 L/s. Prior to sample collection the water column was exchanged 2.5 times. Pumped groundwater was collected in sterile 10 L Nalgene^®^ containers in triplicates and transported back to the laboratory for immediate filtration. Sampling site 2 is a drinking water supply well in Entringen with a permanent pumping system, where a tap at the well head was used for sampling. Prior to sampling, the production well was operated for at least 1 h to maintain steady state conditions. Samples were collected in triplicates in sterile 10 L Nalgene^®^ containers, transported back to the laboratory and immediately filtered. In the laboratory, groundwater samples were filtered sequentially through 8 μm (Millipore, TETP04700), 0.4 μm (Millipore, HTTP04700), and 0.2 μm (Millipore, GTTP04700) polycarbonate filters. The filters were frozen at −20°C until further analysis. DNA was extracted from the 0.2 μm filters using the FastDNA spin kit (MP Biomedicals, Santa Ana, CA, United States) according to the manufacturer’s instructions.

#### Soil

Topsoil (0–10 cm depth) was collected using a sterile ethanol-washed spatula into sterile Corning^TM^ Falcon 50 mL Conical Tubes. Samples were transported at ambient temperature back to the laboratory (within 2 h) and frozen at −80°C. DNA was extracted according to Lueders et al. (2004).

#### Sediment

Sediment was collected using a sterile ethanol-washed stainless steel corer with an inner diameter of 4 cm. Subsamples from 5 cm depth were removed carefully from the core using a sterile autoclaved stainless-steel spatula and placed into sterile Corning^TM^ Falcon 50 mL Conical Tubes before being frozen immediately on dry ice in the field. DNA was extracted from 0.5 g (wet weight) according to Lueders et al. (2004).

#### River Water

River water samples were collected in triplicates into sterile 10 L Nalgene^®^ canisters. Sample containers were held below the water surface (at ∼20 cm depth) and transported back to the laboratory for immediate filtering (i.e., within maximum 8 h after retrieval) through a 0.2 μm polyethersulfone filter (Steritop; EMD Millipore). Filters were frozen at −20°C until DNA extraction. DNA was extracted from the filters using the FastDNA spin kit (MP Biomedicals, Santa Ana, CA, United States) according to the manufacturer’s instructions.

Further details such as sample names, sampling coordinates, and sampling dates can be found in the Supplementary Table 2.

### 16S rRNA Gene Amplicon Sequencing

Bacterial and archaeal 16S rRNA genes were amplified using universal primers 515F: GTGYCAGCMGCCGCGGTAA (Parada et al., 2016) and 806R: GGACTACNVGGGTWTCTAAT (Apprill et al., 2015) fused to Illumina adapters. PCR mixtures for amplification contained (per 25 μl reaction): 0.5 μl of each primer (515F and 806R with Illumina tags; 10 μM stock concentration), 12.5 μl of 2× KAPA HiFi Hotstart Readymix (Kapa Biosystems, Inc., Wilmington, MA, United States), 0.5 μl BSA (10% stock solution), 10 μl of RNAse/DNAse-free water and 1 μl of template. The thermal profile used was: 3 min at 95°C, 27 cycles of 95°C 30 s, 55°C 30 s, 72°C 30 s and 5 min at 72°C. Subsequent library preparation steps (Nextera, Illumina) and 250 bp paired-end sequencing with MiSeq (Illumina, San Diego, CA, United States) using v2 chemistry were performed by Microsynth AG (Balgach, Switzerland) and between 40,000 to 132,000 read pairs per sample were obtained totaling to 2,368,742 read pairs with 1,166,187,315 nucleotides. Primers were removed from raw sequences and untrimmed sequences were discarded with Cutadapt v1.14 (Parada et al., 2016) wrapped by Trim Galore! v0.4.5 (Felix Krueger)^1^.

### 16S rRNA Gene Amplicon Sequencing Analysis Software

The mock and environmental datasets were analyzed without (Extreme dataset) or with (all other datasets) primer trimming with Mothur, QIIME1, QIIME2, MEGAN as described below. The choice of customized parameters is explained in the Supplementary Material.

For Mothur analysis, Mothur v1.40.5 (Schloss et al., 2009) was used with standard settings following the MiSeqSOP (Kozich et al., 2013), except adjusting the cutoff of the reference alignment to the majority of aligned reads. Briefly, paired-end sequences were merged and only those with maximum eight homopolymers and maximum 275 bp were retained. SILVA v132 alignment was cut to the amplified region (position 11894–25319), and unique merged sequences were aligned to the cut SILVA alignment. The alignment region was refined (Balanced: position 1968–11546, all other: position 1968–11550) and only sequences aligned in that region were retained. Next, unique sequences were pre-clustered allowing for up to 2 nucleotide differences between sequences. Chimeras were removed by VSEARCH. Uncorrected pairwise distances were calculated and finally the sequences were clustered to OTUs at 0.03 (97% similarity) or 0.01 (99% similarity) cutoff and the consensus taxonomy for each OTU was retrieved.

For QIIME1 analysis, QIIME v1.9.1 was applied (Caporaso et al., 2010b) using fastq-join v1.3.1 (Aronesty, 2013) for read merging, PyNAST v1.2.2 (Caporaso et al., 2010a) for alignments, VSEARCH v2.3.4 (Rognes et al., 2016) for OTU picking and chimera detection, uclust v1.2.22 (Edgar, 2010) for taxonomy assignments with python v2.7.13 (van Rossum, 1995) and matplotlib v1.4.3 (Hunter, 2007).

For QIIME2 analysis, primer-free sequences were imported into QIIME2 q2cli v2018.06 (Bolyen et al., 2019), visually inspected with demux^2^, and processed with DADA2 (Callahan et al., 2016) to remove PhiX contamination, trim reads, correct errors, merge read pairs and remove PCR chimeras, or merged with VSEARCH (Rognes et al., 2016) followed by removal of PCR chimeras and Deblur (Amir et al., 2017) to obtain representative ASV sequences. Representative sequences and their abundances were extracted by feature-table (McDonald et al., 2012). A Naive Bayes classifier (Pedregosa et al., 2011) was fitted with 16S rRNA gene sequences extracted from SILVA v132 (Quast et al., 2013) using the PCR primers of the investigated dataset. The representative sequences were classified by taxon using the fitted classifier^3^. QIIME2 plugins were executed with standard parameters, with DADA2 quality settings “- -p-trunc-len-f” and “- -p-trunc-len-r” for Extreme dataset 160 and 120 or for HMP and Balanced datasets with 200 and 120 or for environmental samples with 180 and 180 or with Deblur parameter “- -p-trim-length” 250 for Balanced dataset or 252 for Extreme and HMP datasets. Sequencing data from environmental samples originated from three MiSeq runs that were independently processed by DADA2 and subsequently merged in QIIME2.

For MEGAN analysis, reads were merged using ClipAndMerge v1.7.4 (Peltzer et al., 2016) and merged reads were aligned to SILVA using MALT v0.4.0 (Herbig et al., 2016) with parameters “- -mode BlastN - -alignment Type SemiGlobal - -sparseSAM”. MEGAN v6.10.2 (Huson et al., 2016) assigned taxonomy (taxon path at genus level, all leaves) and abundances (assigned counts) based on MALT alignments.

To facilitate reproducibility and to disseminate bioinformatics applications according to the FAIR principle (Wilkinson et al., 2016) all analysis software for the benchmarks was bundled in containers, using Singularity v2.4.1 (Kurtzer et al., 2017) with Ubuntu 16.04.3 LTS and Conda/Bioconda 4.0.5 (Continuum Analytics, Inc.)^4^ and are publicly accessible. Information how to access these is available in the Supplementary Table 3.

The pipeline nf-core/ampliseq v1.1.0 (Straub and Peltzer, 2019) was executed on environmental data sets using nextflow v19.10.0.5170, Java v1.8.0_112, and singularity v3.0.1 with optional parameters “- -multiple Sequencing Runs” (because the sequencing data originated from three MiSeq runs), “- -trunclenf 180”, “- -trunclenr 180” (to resemble truncation values of QIIME2 with DADA2), “- -classifier_removeHash” (because hash sign in some taxa names lets QIIME2 v2018.6 fail), and a metadata sheet was specified as indicated by the documentation.

### Reference Database

The SILVA v132 database (Quast et al., 2013) of 16S rRNA gene sequences, clustered at 99% similarity, was used as reference database. The used analysis software required specialized files that are indicated in the Supplementary Table 4.

### Statistical Analysis

The F-score was calculated as in Kopylova et al. (2016):

Fscore = 2^∗^precision^∗^recall/(precision + recall)

where precision = (TP)/(TP + FP) and recall = (TP)/(TP + FN), with TP = true positive, FP = false positive, FN = false negative.

On the sequence level, only perfect matches and those with one mismatch to a reference sequence were counted as true positives. However, in the case where multiple ASVs/OTUs matched one reference sequence, only one was counted as expected and all others as unexpected (false positives).

One-way ANOVA followed by Tukey’s multiple comparisons of means was performed in R base v3.4.4 (R Core Team, 2018).

### Plotting

All representative sequences were aligned to reference sequences with blastn v2.2.31+ and a jitter plot based on relative sequence abundances was produced by ggplot2 v2.2.1 (Wickham, 2009) in R v3.4.4 (R Core Team, 2018). The heatmap was done with pheatmap v1.0.8 (Kolde, 2015), the upset plot with UpSetR v1.4.0 (Conway et al., 2017) and the Venn diagram with gplots v3.0.1 (Warnes et al., 2009) in R.

### Diversity Indices and Distances

ASV/OTU sequences were subsequently aligned with Mafft v7.310 (Katoh and Standley, 2013), highly variable positions were masked, an unrooted phylogenetic tree was constructed with FastTree v2.1.10 (Price et al., 2010) and finally rooted by the midpoint of the longest tip-to-tip distance in QIIME2. Shannon’s Diversity Index, Unweighted UniFrac and Bray-Curtis dissimilarity were calculated with the R-package phyloseq v1.22.3 (McMurdie and Holmes, 2013) with the “estimate_richness” or “distance” function using ape v5.1 (Paradis et al., 2004). The Faith’s PD index was calculated with picante v1.7 (Kembel et al., 2010) in R v3.4.4 (R Core Team, 2018). For mock samples, expected alpha-diversity was calculated based on expected sequences and abundances. For environmental samples, Bray-Curtis dissimilarity or Unweighted UniFrac distances were subjected to NMDS (Non-metric Multidimensional Scaling) ordination and combined by Generalized Procrustes Analysis using plyr v1.8.4 (Wickham, 2011) and FactoMineR v1.41 (Husson et al., 2008) and Procrustes Similarity Indices were extracted.

## Results

### Mock Datasets Showed Highest Sensitivity With QIIME1 but Highest Specificity With QIIME2

To evaluate the performance of the 16S rRNA (gene) amplicon sequencing analysis tools, three mock datasets (i.e., Balanced, Extreme, and Human Microbiome Project; HMP) based on samples with known composition were analyzed with Mothur, QIIME1, QIIME2, and MEGAN. First, the number of recovered 16S rRNA gene amplicon sequences (i.e., OTUs or ASVs) were compared to the expected numbers, determined based on the reference sequences and abundances, and used as the basis for subsequent analyses. Only QIIME1, Mothur, and QIIME2 generated sequences that could be compared to defined mock communities. MEGAN did not generate sequences and, therefore, did not allow this comparison. The number of OTUs or ASVs generally overestimated the number of expected unique 16S rRNA gene amplicons for all three datasets (Table 1). Mothur and QIIME1 in particular calculated 10- to 200-fold more sequences than expected (Table 1), with 97% clustering similarity being at the lower end and 99% at the upper threshold. The number of sequences was much better estimated by QIIME2 in combination with DADA2 or with Deblur, however, Deblur underestimated the number of sequences for the Extreme dataset by almost 50%.

**TABLE 1:** Generated sequences (i.e., OTUs or ASVs) for each analysis pipeline (Mothur, QIIME1, and QIIME2) and mock dataset (Balanced, Extreme, HMP).

		Balanced	Extreme	HMP
Species		57^a^	27	21
Unique amplicons		62	35	25
Mothur	99^b^	1681	8009	1556
	97^b^	527	367	546
QIIME1	99^b^	1474	5545	4161
	97^b^	1002	1411	2826
QIIME2	DADA2^c^	89	50	73
	Deblur^c^	74	18	35

*^a^57 species but only 52 distinguishable with the sequenced amplicon. ^b^Similarity (%) at which sequences are clustered into operational taxonomic units (OTUs). ^c^ASV calling software.*

**FIGURE 1:**
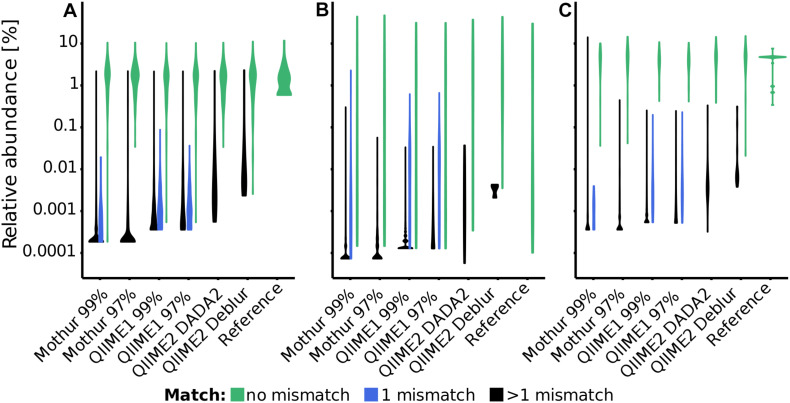
Distribution of relative abundance of sequences (i.e., OTUs or ASVs) and their distance to reference sequences for each pipeline and dataset [**(A)**: Balanced, **(B)**: Extreme, **(C)**: HMP]. Distribution of sequences with perfect match to a reference sequence are depicted in green, with one mismatch in blue and with more than one mismatch in black. In this Violin plot, the width (*x*-axis) of the shapes corresponds to the fraction of sequences within that distribution, i.e., the thicker the line the more sequences were recovered at a particular relative abundance.

The accuracy of recovered 16S rRNA (gene) sequences and the relative sequence abundance is of particular interest for subsequent taxonomic classification or phylogenetic tree construction as well as for a realistic representation of microbial community composition. In the three mock datasets, QIIME1 showed highest sensitivity and recovered 83 to 94% of the reference sequences, closely followed by QIIME2 using DADA2 with 71 to 95% recovered sequences (Figure 1 and Supplementary Table 5). The lowest sensitivity with only 43% (15 of 35 total; Supplementary Table 5) recovered sequences was found for QIIME2 using Deblur while processing the Extreme dataset, where mainly low abundant sequences failed to be recovered (Figure 1B). QIIME2 in combination with Deblur was most specific for all datasets with only 3 to 18 unexpected sequences, followed by QIIME2 in combination with DADA2 that produced 25 to 50 sequences that did not perfectly match to a reference sequence. However, of the 30 unexpected sequences found by QIIME2 with DADA2 in the Balanced dataset (Figure 1 and Supplementary Table 5), 13 were reported by all pipelines, 10 were detected by all but one pipeline, and only three sequences were found exclusively by QIIME2 with DADA2 and no other pipeline. QIIME1 and Mothur detected at least 10 times more unexpected sequences than QIIME2 (Figure 1 and Supplementary Table 5). Using a 99% similarity threshold for OTUs with Mothur or QIIME1 did not improve sensitivity compared to the 97% similarity threshold but was highly detrimental to specificity by increasing the number of unexpected sequences by 50 to 2,000% (Figure 1 and Supplementary Table 5). At the time of conducting this study, Mothur’s MiSeq Standard Operating Procedure (SOP) advertised the possibility to produce ASVs before clustering into OTUs (but after pre-clustering with 2 bp distance). Because increasing the sequence similarity threshold from 97 to 99% nucleotide identity for clustering decreased performance, excluding clustering (i.e., 100% clustering similarity threshold) to produce ASVs was expected to under perform and was therefore omitted for the analysis with Mothur.

The F-score (Kopylova et al., 2016), that is the harmonic mean of precision (detected reference sequences to all *predicted* sequences) and recall (detected reference sequences to all *reference* sequences), was much higher (i.e., better) for QIIME2 than for all other pipelines (excluding MEGAN) (Supplementary Table 5). The F-score was mostly driven by the precision, unexpected sequences (false positives) mostly occurred below 1% relative sequence abundance, but the majority of unexpected sequences occurred below 0.001% to 0.1% abundance, depending on the pipeline (with QIIME1 at the higher end) and the dataset. For example, the Balanced dataset analyzed with QIIME1 had the majority of non-perfect matching sequences present at less than 0.01% relative sequence abundance but the Extreme dataset at less than 0.1% relative sequence abundance (Figure 1). Concordantly, the F-score substantially improved for Mothur and QIIME1 results when applying relative abundance cutoffs, but increased much less for QIIME2 (Supplementary Figure 1).

### Taxonomic Representation of the Mock Datasets Was Best Resembled by QIIME2

**FIGURE 2:**
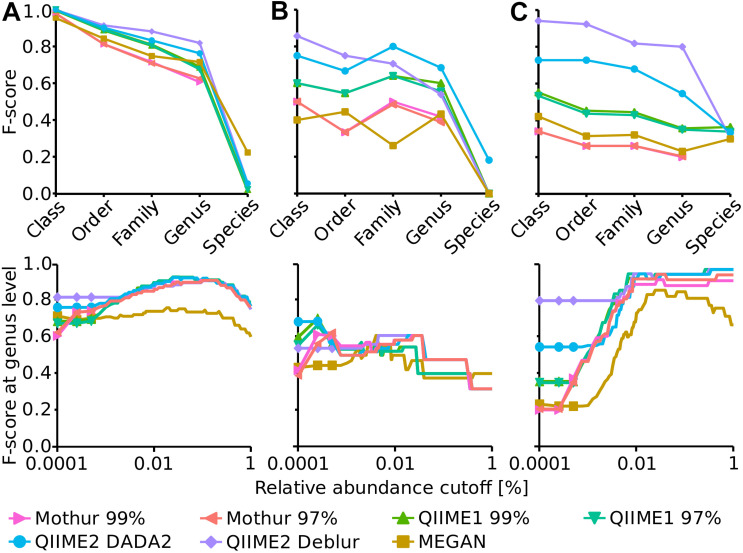
Upper panels: F-score at several taxonomic levels (i.e., class, order, family, genus and species) for Balanced **(A)**, Extreme **(B)**, and HMP **(C)** mock datasets. Lower panels: F-score at genus level dependent on a relative abundance cutoff (up to 1%).

To assess the accuracy of the workflows until taxonomic classification (i.e., including errors from OTU/ASV generation), the F-score was calculated for several taxonomic levels (i.e., class, order, family, genus and species). Taxonomic classification varied substantially among pipelines, for instance using the HMP dataset, F-scores from 0.2 (Mothur) to 0.8 (QIIME2 in combination with Deblur) were generated at genus level (Figure 2). Generally, QIIME2 had either close to the highest or the highest F-score of all four analysis pipelines in all datasets (Figure 2), meaning that the compromise between precision and recall was best for QIIME2. Among all investigated pipelines, F-scores were similar for the Balanced dataset, but QIIME1 and QIIME2 achieved best results (i.e., highest F-scores) for the Extreme datasets and QIIME2 for the HMP dataset above species level, i.e., genus level and higher. This difference was mainly driven by the superior precision of QIIME2 that was determined for all investigated datasets and for all taxonomic levels above species level. QIIME2’s Deblur outperformed DADA2 slightly on the Balanced dataset and more pronounced with the HMP dataset but had a lower F-score on family and genus level with the Extreme dataset due to Deblur’s higher precision but lower recall.

Mothur and MEGAN achieved the lowest F-score for all taxonomic levels. In order to optimize the taxonomic classification with MEGAN, its 16S Percent Identity Filter was enabled and taxonomic assignments were projected to respective ranks, however, this did not improve taxonomic classification substantially compared to default settings. In fact, until genus level, the F-score for all three mock datasets were almost identical to those calculated with default settings but the species classifications were improved to the best values among all pipelines for Balanced and Extreme datasets, however, worsened for HMP data.

Generally, F-scores at species level were very low compared to higher taxonomic ranks. Mothur did not attempt to annotate the species rank at all unlike QIIME1, QIIME2, and MEGAN. Overall, the HMP dataset had the highest species annotation score of the three datasets with very similar values for all four analysis pipelines that annotated species (Figure 2).

The impact of relative abundance cutoffs on the F-score at genus level was investigated exemplary for all taxa levels (Figure 2, bottom panels). Genus level was chosen because it was the lowest taxonomic level that produced reasonable results (i.e., species level had much lower F-score) and is also often the taxonomic level of choice when microbial communities are investigated with amplicon sequencing. The relative abundance cutoffs were either applied to the OTU/ASV table (Mothur, QIIME1 and QIIME2) or to the taxonomic classification (MEGAN). Relative abundance cutoffs had very different effects on the F-score for the three mock communities. The F-score of all methods slightly increased until ca. 0.1% abundance cutoff for the Balanced dataset but decreased afterward because of the loss of true positive genera. For the Extreme dataset, the F-score improved for the OTU-producing methods Mothur and QIIME1 with a very low abundance cutoff (0.00025%) but then steadily decreased for all methods due to the low abundance of some expected genera that were lost (i.e., increasing false negatives). The maximum F-score of 0.68 was reached by QIIME2 with DADA2 with no abundance cutoff. The F-score for the HMP dataset substantially increased for all methods with increasing relative abundance cutoff (i.e., up to 0.97) due to improving precision (all methods had a relatively high false positive rate) and because all expected genera were at high abundance (>0.5%).

### Alpha-Diversity Indices of the Mock Datasets Were Approximated Most Closely by QIIME2

**FIGURE 3:**
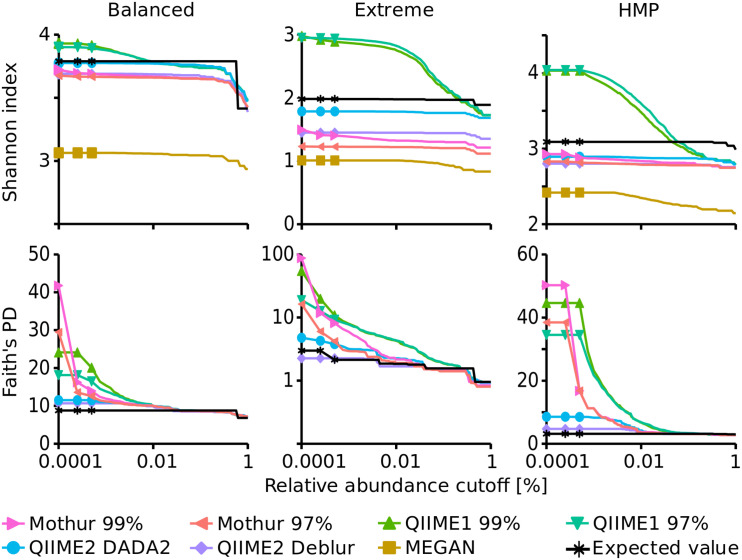
Alpha diversity indices dependent on a relative abundance cutoff (up to 1%). Upper panels: Shannon index, lower panels: Faith’s Phylodiversity; note the logarithmic *x*-axis and for Faith’s PD of the Extreme dataset the logarithmic *y*-axis.

The Shannon index (Shannon, 1948) that determines how many different types of species or sequences (i.e., OTUs or ASVs) are present in a sample (richness) and how evenly these are distributed (evenness) followed the expected trend for Mothur and QIIME2, i.e., the diversity decreased from Balanced (expected: 3.79) to HMP (expected: 3.09) to Extreme datasets (expected: 1.98). However, QIIME1 surprisingly led to a higher Shannon index for the HMP dataset (4.04) than for the Balanced dataset (3.90). The Shannon index of the Balanced dataset was relatively independent of the analysis pipeline and varied only slightly from 3.68 (Mothur 97%) to 3.93 (QIIME1 99%) when excluding the outlier MEGAN (3.06). But, for the HMP and Extreme datasets the pipelines came to different results with 2.42 (MEGAN) to 4.04 (QIIME1 99%) for the HMP dataset and 1.01 (MEGAN) to 2.98 (QIIME1 99%) for the Extreme dataset. Generally, QIIME1 overestimated the Shannon index for all mock datasets, while QIIME2 and Mothur slightly underestimated the values and MEGAN heavily underestimated the diversity in all datasets by 20 to 60% (Figure 3). Enabling MEGAN’s 16S Percent Identity Filter shifted the calculated Shannon index closer to the expected values for Extreme data but further away for the other two datasets compared to default settings. However, the expected Shannon indices were calculated on sequence level and MEGAN used genera abundance estimates instead of fine-grained OTU or ASV sequences and therefore was not able to closely resemble the expected numbers.

Overall, Shannon alpha diversity indices were most accurately reproduced by QIIME2 in combination with DADA2 though this method underestimated Shannon diversity on average by 6% (0.3 to 10% for the three datasets).

Faith’s Phylodiversity (PD) index (Faith, 1992) that is a qualitative measure of the sum of the phylogenetic branch lengths covered by a sample was also best resembled by QIIME2 with almost 2-fold overestimation on average (1.3- to 2.7-fold for the three datasets) but strongly overestimated by QIIME1 (2- to 18-fold) and Mothur (3.5- to 29-fold). The estimates improved with increasing relative abundance cutoff and resembled the expected values very closely, ranging from 0.9- to 1.2-fold of the expected values when only sequences above 0.1% abundance were considered (Figure 3).

### Poor Agreement in Recovery of 16S rRNA Gene Amplicon Sequences and Taxa of Environmental Samples Between Analysis Methods

**FIGURE 4:**
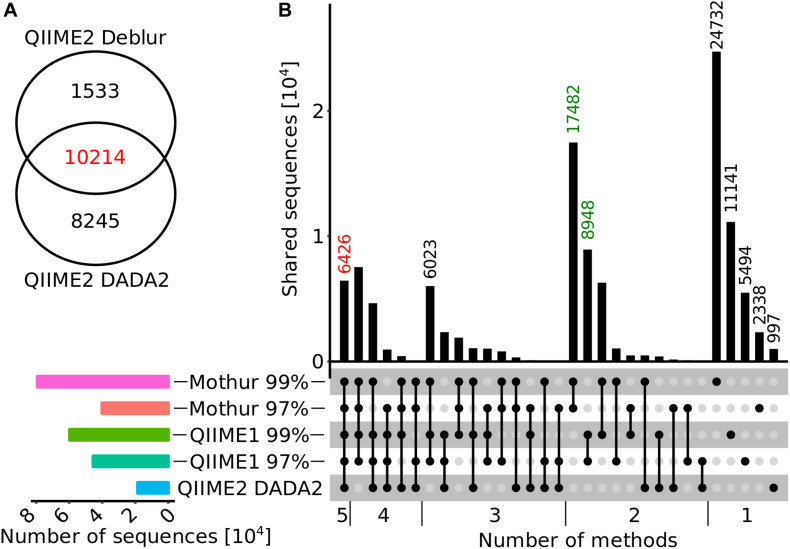
Overlap diagrams for ASV or OTU sequences. **(A)** Venn diagram for QIIME2 with DADA2 and Deblur sequences that were trimmed to 250 bp for comparison. **(B)** Horizontal bar plot (bottom, left panel) represents the number of sequences reported for each method (from top to bottom: Mothur 99%, Mothur 97%, QIIME1 99%, QIIME1 97%, QIIME 2 with DADA2) and matrix (bottom, right panel) indicates methods that are part of an intersection with (connected) black filled circles. The vertical bar graph (top, right panel) represents the number of shared sequences (intersection size) for each intersection. In the vertical bar graph, the number of sequences calculated by all methods are highlighted in red and sequences only found within QIIME1 or Mothur in green. QIIME2 with Deblur was excluded in this figure because its ASVs have a different length (250 bp) than that expected of OTUs/ASVs of all other methods (>98% above 250 bp).

Environmental samples typically have a more complex microbial community than mock datasets and, therefore, we selected 24 samples from diverse habitats (groundwater, soil, river sediment, and river water) for analysis using 16S rRNA gene sequencing to investigate whether results differed between the investigated pipelines. First, the numbers of reported sequences (i.e., OTUs and ASVs) and unique genera for each analysis pipeline were compared to investigate whether the trend observed in the mock datasets was also evident in the environmental samples, since measures such as estimates of community diversity or clustering distance strongly depend on sequence or taxa count. Total sequence numbers, e.g., OTUs or ASVs, across all samples varied from 11,747 with QIIME2 and Deblur to 79,326 with Mothur. This was a similar trend compared to the analysis of the mock datasets, where QIIME2 with Deblur and DADA2 produced the lowest amount of sequences (ASVs) while QIIME1 and Mothur counted the highest number of sequences (OTUs). Most sequences (10,214 ASVs) computed by Deblur (87%) or DADA2 (55%) were identical (Figure 4A). In addition, there was relatively large overlap between Mothur, QIIME1, and QIIME2 with 6,426 (only 6% of total, but 35% of QIIME2 with DADA2) identical sequences (Figure 4B), whereas 62,755 sequences (55%) were shared by at least two but not all pipelines and 44,702 sequences (39%) were not shared at all. 17,482 and 8,948 OTU sequences overlapped within Mothur or QIIME1 with varying similarity cutoffs (i.e., 97 or 99%), respectively, and both analysis pipelines produced 1.3- to 2-fold more OTU sequences with the 99% similarity cutoff than with 97%, in line with the findings of the mock community analysis that produced 1.5- to 22-fold more OTUs with 99% similarity cutoff.

**FIGURE 5:**
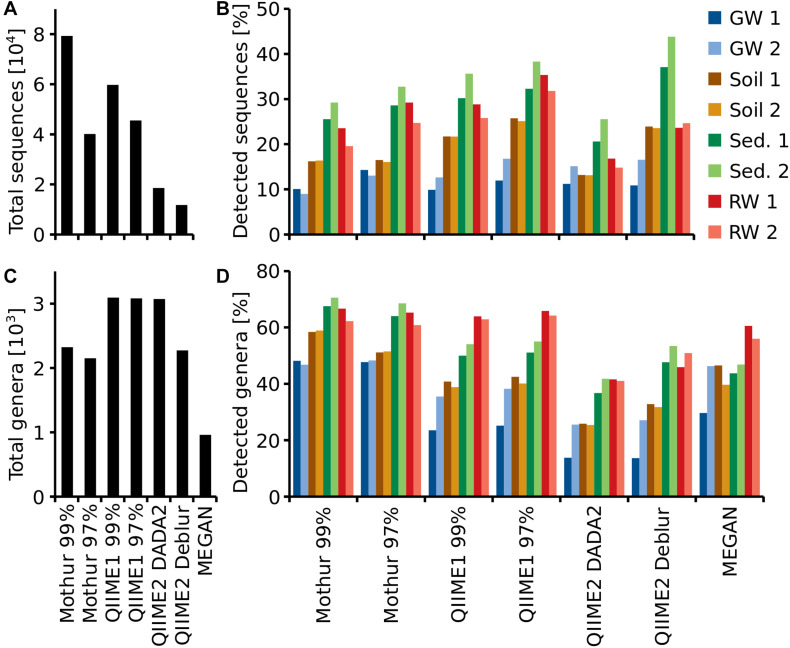
Total number of recovered sequences (OTUs or ASVs) **(A)** and genera **(C)**, for each pipeline and percentage of total sequences **(B)** and genera **(D)** detected at each sampling site. For each habitat (groundwater, soil, sediment or river water), two sampling sites (1, 2) were investigated.

Reported genera derived from the up to 8-fold different sequence counts (11,747 to 79,326, Figure 5A) varied much less, from around 2,200 using Mothur or QIIME2 with Deblur to almost 3,100 using QIIME1 or QIIME2 with DADA2. MEGAN reported 961 genera, which was by far the lowest number (Figure 5B). All pipelines recovered the highest number of sequences from sediment site 1 (26 to 44% of total) and least sequences in groundwater site 1 (QIIME1: 10 to 12% and QIIME2: 11%) or site 2 (Mothur: 9 to 13%). Most genera, however, were found in sediment site 2 (Mothur: 69 to 71% and QIIME2: 42 to 53%) or river water site 1 (QIIME1: 64 to 66% and MEGAN: 61%). But generally, all pipelines identified more sequences and genera in sediment and river samples than in groundwater and soil samples (Figure 5).

QIIME2 with DADA2 found on average the same number of ASVs per sampling site as QIIME2 with Deblur. However, QIIME2 with DADA2 found almost twice the number of ASVs overall compared to QIIME2 with Deblur (Figure 5A). Thus, more unique sequences were found per sampling site with DADA2 while Deblur found the same ASVs at multiple sampling sites (Figures 5A,B). The same was also observed at genus level (Figures 5C,D).

**FIGURE 6:**
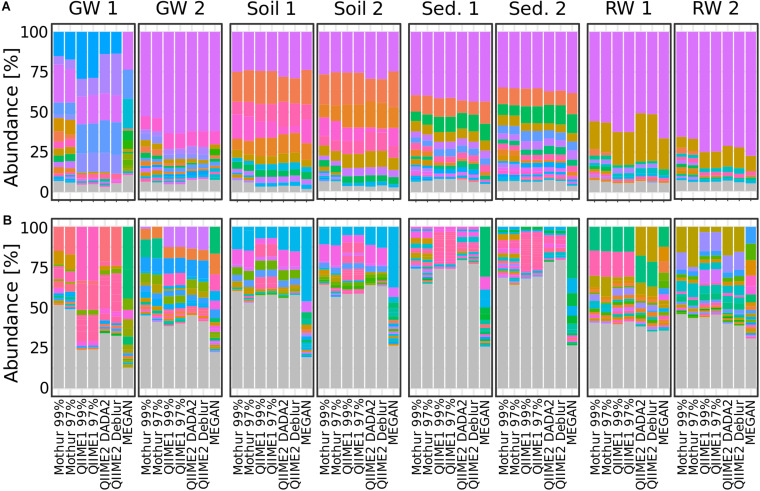
Bar plots showing relative taxa abundance averaged over triplicates at **(A)** phylum or **(B)** genus level for all habitat sampling sites and analysis methods. Genera or phyla are shown in the same colors throughout the figure. Gray are taxa <1% abundance. GW, groundwater; Sed., sediment; RW, river water; 1 and 2 indicate sampling sites.

To see how the analysis methods differed in the resulting microbial community composition, the relative abundance at phylum and genus level was compared. Generally, at phylum level the community composition at each individual site was very similar regardless of the pipeline with one exception; at groundwater site 1, the community composition showed large differences between all tested pipelines at phylum level (Figure 6A and Supplementary Figure 2). At genus level, however, dramatic differences in the resulting community composition were observed for all pipelines. For example, while the community composition at phylum level at river water site 2 was fairly consistent between all pipelines, the results at genus level were substantially different to the extent that the most abundant genera were not detected across all pipelines (Figure 6B and Supplementary Figure 3). Some similarities were observed between Mothur and QIIME pipelines, for example, the community composition at genus level in soil site 1 differed only slightly. In contrast, the differences between MEGAN and other pipelines varied substantially.

**FIGURE 7:**
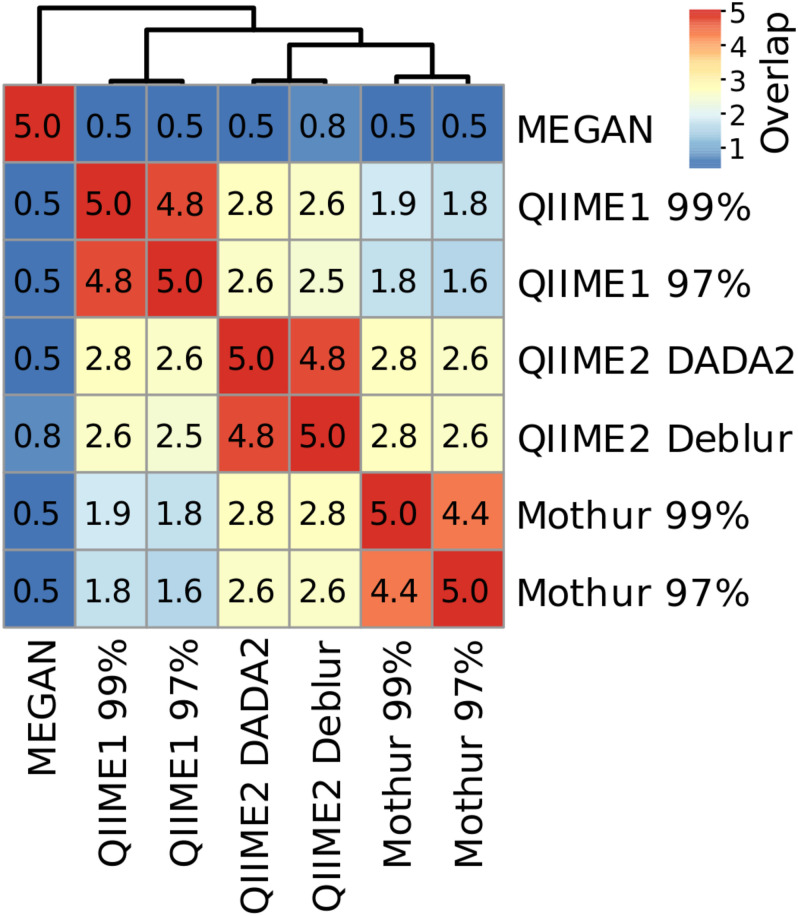
Heatmap and number of shared taxa among the top five most abundant genera for each pipeline averaged across sampling sites. Genus names and their relative abundance can be found in Supplementary Table 6.

Because dominant microbial taxa might have important functions at specific sites, the five most abundant taxa at genus level were compared for each sampling site and analysis method. QIIME1 with 99% or 97% similarity threshold identified always the exact same four most abundant taxa but differed twice in the fifth most abundant taxa (soil site 2 and sediment site 1). QIIME2 using DADA2 agreed almost perfectly with QIIME2 using Deblur, differing only in two sampling sites: sediment site 2 with DADA2, unclassified member of *Acidobacteria* subgroup 6, vs. with Deblur, unclassified *Nitrososphaeraceae;* river water site 2 with DADA2, *Hydrogenophaga*, vs. with Deblur, *Pseudomonas* (Supplementary Table 6). Very little difference between the similarity thresholds of 99% and 97% was observed for Mothur which had on average more than four identical taxa in the top five most abundant ones across sampling sites (Figure 7 and Supplementary Table 6). MEGAN had lowest agreement with all other methods and none or maximum two reported genera per sampling site matched those found using the three other methods (Figure 7 and Supplementary Table 6). On average, up to two overlapping taxa were observed by Mothur compared to QIIME1, 2.6 taxa were shared by QIIME1 and QIIME2, and 2.7 overlapping taxa were found by Mothur compared to QIIME2.

Differences were also observed in the consistency of the five most abundant genera between analyses depending on sampling site. For example, at soil site 1, the average number of overlapping most abundant genera was 3.0. Conversely, sediment site 1 had the lowest average number of overlapping genera with 1.2, showing a clear difference in the community composition results, depending on the analysis method (Supplementary Table 6). However, across all samples, even when the same genera were found, the abundance and the order of abundance varied with the analysis method. For example, river water site 2 analyses showed that (except for MEGAN) *Rhodoferax*, *Malikia* and *Flavobacterium* were consistently present in the top five most abundant genera. However, of these three taxa, according to Mothur with 97% similarity threshold, *Malikia* had highest abundance (9%) and *Rhodoferax* the lowest (3%), while according to all other methods *Rhodoferax* had the highest abundance (10–12%) and *Flavobacterium* the lowest (3–4%). Additionally, the relative abundance for *Malikia* varied from 4% (QIIME1) to 9% (Mothur). On the other hand, most analyses agreed about the order of abundance of the three most abundant taxa at groundwater site 2 which was dominated by *Gallionellaceae* (Mothur) or *Sideroxydans* (QIIME1 and 2), belonging to the family *Gallionellaceae*, followed by *Polaromonas* and *Acinetobacter* (when disregarding the unassigned sequences in QIIME1). In contrast to all other analyses, MEGAN reported an unclassified taxon, *Acinetobacter*, and *Candidatus* Omnitrophica as the three most abundant taxa at groundwater site 2 (Supplementary Table 6).

The difference of reported abundant taxa probably has implications for the interpretation of important biogeochemical cycles. For instance, two of the dominating genera at the investigated river water sites, *Sphaerotilus* and *Agitococcus*, were only reported when using QIIME1 or QIIME2 (Supplementary Table 6). At river water site 2, *Sphaerotilus*, potentially involved in dissimilatory nitrate reduction to ammonium (DNRA) or partial denitrification (nitrate reduction to nitrous oxide) (Kanehisa and Goto, 2000; Gridneva et al., 2011), was reported at 8% by QIIME1 but remained undetected when analyzed with QIIME2, Mothur, or MEGAN. At river water site 1, *Agitococcus lubricus*, potentially involved in nitrite ammonification and sulfate reduction (Franzmann and Skerman, 1981; Chen et al., 2018), was reported by QIIME1 and QIIME2 at 2 to 3% but not by Mothur and MEGAN. However, it remains uncertain which results present reality more accurately. It is, however, more plausible to follow the results of QIIME1 with its highest sensitivity but also high proportion of false detections or QIIME2 with its highest accuracy in mock datasets than to pursue what Mothur or MEGAN could not detect.

### Diversity Estimates in Environmental Samples Varied Among Tools

**FIGURE 8:**
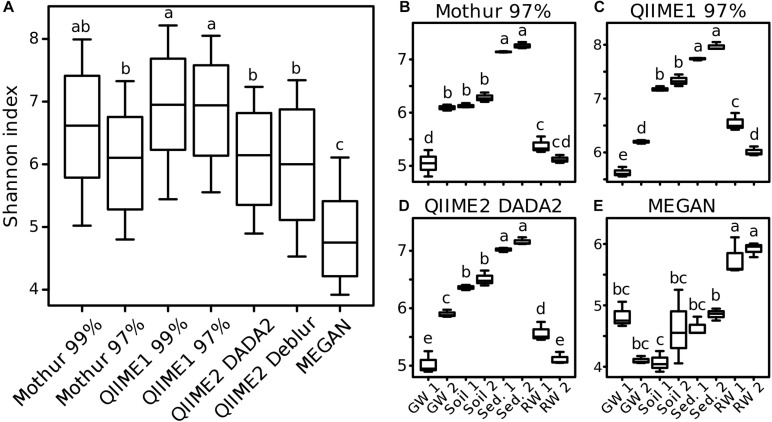
Comparison of alpha-diversity indices for environmental samples. For each analysis pipeline, Shannon indices were calculated either on all samples combined **(A)** or each sampling site separately **(B–E)**: GW, groundwater; Sed., sediment; RW, river water; 1 and 2 indicate sampling sites. Significance of differences between samples (One-way ANOVA followed by Tukey multiple comparisons of means, *p* < 0.05) is marked by lowercase letters so that samples that share at least one letter are not significant different (e.g., “a” and “ab”) but samples that do not share a letter are significant different (e.g., “a” and “b”).

Differences in diversity estimates between methods might be caused by the substantial differences of the number of OTUs and ASVs, their sequences, and taxonomic classifications (even of high abundant taxa). To investigate the comparability of within-sample diversity measures (alpha-diversity), the Shannon index was calculated for results of each analysis pipeline. Generally, across all samples, QIIME2 with DADA2 and with Deblur reported similar values (±1%) to Mothur with 97% similarity threshold for the Shannon index. In comparison, QIIME1 and Mothur with 99% similarity threshold had 13 and 9% higher values, respectively, while MEGAN calculated 20% lower values (Figure 8A). The trend seemed very similar among all analysis pipelines except for MEGAN (Figures 8B–E), with descending diversity from sediment, to soil, to groundwater, and river water, although there were differences in absolute Shannon diversities. In contrast, MEGAN reported river water having the highest Shannon diversity followed by all other habitats (Figure 8E). One exception was groundwater sampling site 2 (GW 2, Figures 8B–D) that had a similar Shannon index to both soil sampling sites in the analysis with Mothur (Figure 8B), but was significantly different to soil with QIIME1 and QIIME2. GW 2 had a similar Shannon index to river water (RW 2) with QIIME1 (Figure 8C), but was significantly different to river water sampling sites with QIIME2 (Figure 8D).

**FIGURE 9:**
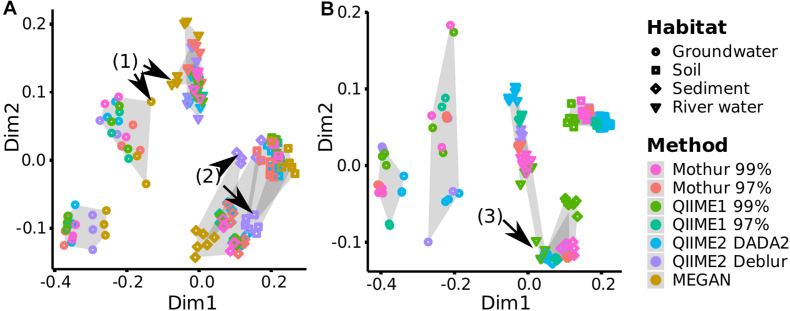
Comparison of beta-diversity plots for environmental samples. For each analysis pipeline, **(A)** Bray-Curtis dissimilarity (quantitative) or **(B)** Unweighted UniFrac distances (qualitative and phylogenetic) were subjected to NMDS (Non-metric Multidimensional Scaling) ordination and combined by Generalized Procrustes Analysis (NMDS stress values <0.06, Supplementary Table 7). Gray shading marks sampling sites, e.g., all three replicates analyzed by the different pipelines from one sampling site are connected by gray background. Black arrows highlight differences discussed in the text.

To investigate if each pipeline allowed similar sample groupings, distances based on OTUs (Mothur and QIIME1), ASVs (QIIME2) or taxa (MEGAN) abundance between samples (beta-diversity) were measured. Overall, distances and groupings were similar across pipelines and all of them allowed the separation of habitats except MEGAN, where it was not possible to distinguish between samples from river water and groundwater as clearly as for other pipelines (Figure 9A, arrow 1). Also, the two groundwater sites clustered separately by all pipelines (Figure 9).

In terms of consistency between pipelines, QIIME2 with Deblur exchanged placement of sediment and soil samples on the Bray-Curtis (Bray and Curtis, 1957) dissimilarity plot compared to other pipelines but cluster separation of these two habitats remained stable (Figure 9A, arrow 2). QIIME2 with Deblur and MEGAN had on average the lowest Procrustes Similarity Indices (Sibson, 1978) with 0.88 and 0.90, respectively, compared to other methods (>0.96; Supplementary Figure 4A) meaning that their plots were less similar. Calculation of Unweighted UniFrac (Lozupone et al., 2007) requires sequences (i.e., ASVs or OTUs) so that MEGAN was excluded from the following comparison: QIIME1 with 99% similarity threshold placed the samples from a river site and the sediment samples differently (Figure 9B, arrow 3), while QIIME2 in combination with DADA2 had slightly shifted placement for river and soil samples, resulting in a slightly rearranged plot for both methods compared to all other pipelines (Figure 9B), corroborated also by the Procrustes Similarity Indices (on average, QIIME1 with 99% similarity threshold: 0.90, QIIME2 with DADA: 0.91, all other methods: >0.95; Supplementary Figure 4B).

### The Reproducible and Easy-to-Use Pipeline nf-core/ampliseq Wraps QIIME2 With DADA2

Finally, we implemented a high throughput pipeline named “nf-core/ampliseq” (doi: 10.5281/zenodo.1493841) using QIIME2 with DADA2 as center piece for reproducible analysis of 16S rRNA (gene) amplicon sequencing data and applied it to the environmental samples. Overall, nf-core/ampliseq produced very similar results to QIIME2 with DADA2 and reported identical ASV numbers and sequences. Subsequently, 244 ASVs annotated as mitochondria or chloroplasts were removed by nf-core/ampliseq because these sequences are typically considered unwanted. Filtering these ASVs in nf-core/ampliseq is default but can be adjusted or even omitted.

Using nf-core/ampliseq, the five most abundant genera were in all sampling sites identical to those found using QIIME2 with DADA2 and relative abundances deviated by less than 0.1 percentage point. Shannon’s Diversity Indices reported by nf-core/ampliseq followed the same order compared to that calculated with R package phyloseq based on abundance tables by QIIME2 with DADA2 (Figure 8) but was 40% higher (sediment 10.2, soil 9.2, river water 7.6, groundwater 7.9) because nf-core/ampliseq calculated the values using logarithmic base 2 (QIIME2 implementation with scikit-bio) (The scikit-bio development team, 2020), while phyloseq (calling R package vegan’s diversity function) was using the natural logarithm (base e) (Oksanen et al., 2018). The logarithmic base for Shannon’s Diversity Index is not fixed in the original publication and there is no canonical way to calculate it (Shannon, 1948). Community differences visualized with beta-diversity distances (unweighted UniFrac, Bray-Curtis) in the nf-core/ampliseq showed that habitats were separated significantly (BH-corrected pairwise PERMANOVA with 999 permutations, *q* ≤ 0.01) but differences between sampling sites had a higher uncertainty (0.11 < q < 0.13), very similar to findings with QIIME2 with DADA2 (Figure 9). In summary, the differences between nf-core/ampliseq and QIIME2 with DADA2 are only marginal and are caused by improved data handling by nf-core/ampliseq or different (however, neither better nor worse) tools.

## Discussion

Here we compared 16S rRNA (gene) sequence analysis pipelines and aim at identifying the best suited bioinformatics method to date to analyze environmental microbial communities based on high-throughput DNA- or RNA-based 16S rRNA (gene) amplicon sequencing data. Therefore, mock communities and environmental samples from a range of contrasting habitats with differing geochemical conditions (e.g., redox and nitrate concentrations) were analyzed with popular analysis pipelines, i.e., Mothur, QIIME (version 1 and 2) and MEGAN. We found that (i) QIIME2 results reflected reality most accurately using mock communities, that (ii) interpretations of microbial studies were biased by the analysis method regarding sequence recovery, taxonomic identification and diversity measures and (iii) we implemented a high-quality analysis workflow using the lessons learned in this study.

### The Best Compromise of Sensitivity and Specificity by QIIME2

Generally, Mothur and QIIME1 recovered almost all 16S rRNA gene amplicon sequences and genera but the number and abundance of false positives was relatively high, so that sometimes the true positive sequences were buried underneath false positives. Removing sequences with low relative abundance, e.g., <0.1%, improved the results for QIIME1 and Mothur but had the adverse effect of removing low abundant, expected sequences. Additionally, there was no general advisable abundance cutoff for Mothur or QIIME1 and different datasets had the optimal balance of precision and recall (i.e., F-score) at different relative abundance cutoffs, probably due to data quality. Therefore, it seemed not practical to choose a general abundance cutoff for these tools when analyzing non-mock samples. Low abundant sequences and taxa might be interesting in some studies, e.g., when a group of low abundant microorganisms is performing a crucial step in the biogeochemical cycle, such as carbon and nitrogen cycling or sulfate reduction (Musat et al., 2008; Pester et al., 2010; Jousset et al., 2017), and therefore removing them might be undesirable. QIIME2 using Deblur suffered from relatively low recall (several sequences or taxa were not detected) but had highest precision (a low number of additional false sequences or taxa was detected) which was similar to findings observed by Nearing et al. (2018). A recent study found that DADA2 had difficulties finding low abundant variants and produced few but high abundant false positives (Hathaway et al., 2017), although we did not observe this finding. On the contrary, QIIME2 using DADA2 showed high recall and high precision. However, in the Balanced mock dataset QIIME2 found highly abundant, unexpected sequences, but because these were in the majority (23 of 30 total) also found by all or all but one other method, we assumed these were true sequences not present in the reference database. Essentially, perfect results were not obtained by any method but DADA2 in combination with QIIME2 seemed the best compromise of sensitivity and specificity (Table 2).

**TABLE 2:** Summary of strengths and weaknesses of tested pipelines. All values are means (± standard deviation) of the analyses of three mock datasets.

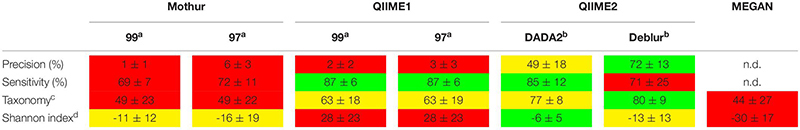

*n.d., not determined by this method. ^a^Similarity (%) at which sequences were clustered into operational taxonomic units (OTUs). ^b^ASV calling software. ^c^F-score on family level in percent. ^d^In % deviation; negative numbers mean underestimation, positive numbers mean overestimation. Colors denote what methods performed best, i.e. green means best, yellow intermediate, and red worst.*

Taxonomic annotation depends on the amplicon region (Kozich et al., 2013), reference database, and the classifier (Almeida et al., 2018). The reference database used in this study was SILVA v132 with 16S rRNA gene sequences dereplicated at 99% similarity, meaning it contained combined taxa with ≥99% similar 16S rRNA gene sequences and thereby reduced the computational requirements. However, it also decreased taxonomic resolution. The mock datasets used here contained sequences of the 16S rRNA gene V4 region with a length of 250 to 254 bp. The choice of the amplified region also restricts taxonomic resolution, e.g., the *Enterobacteriaceae* family and the *Clostridiales* order are known to be poorly resolved using these short V4 amplicons (Jovel et al., 2016) and the resolution at phylum level is lower than sequencing the whole 16S rRNA gene (Yang et al., 2016). But even when using full-length 16S rRNA gene analysis, some related but distinct microorganisms can remain unresolved. For instance, five *Streptomyces* species with identical 16S rRNA gene sequences were shown to have phenotypic, microscopic, genetic and genomic differences (Antony-Babu et al., 2017). Overall, our study showed that species level seemed too biased to be trusted for taxonomic classification. This is in agreement with earlier studies that found species classification unreliable especially for uncharacterized species (Bokulich et al., 2018; Edgar, 2018) but taxonomic classification at genus level was more accurate.

### The Choice of the Analysis Pipeline Affects the Outcomes of Studies

The difference in the number and the quality of recovered 16S rRNA gene amplicon sequences and their further taxonomic classification among pipelines also caused deviations in data interpretation. For instance, the sampling site with the highest microbial diversity among the investigated environmental samples (i.e., groundwater, soil, sediment or river water sites) differed depending on the analysis pipeline. Additionally, differences in microbial diversity estimates led to dissimilar interpretations depending on the analysis pipeline. The choice of the analysis pipeline affected the outcome of our study including interpretations of taxa involved in certain biogeochemical cycles and, thus, special care needs to be taken when interpreting results, particularly when dealing with highly diverse environments.

### Sequence Recovery

Overall, the accuracy of sequence recovery of QIIME2 indicated that this pipeline was the best basis for further downstream analysis and data interpretation. This was due to denoising (i.e., DADA2, Deblur) that performed better than OTU clustering (i.e., Mothur, QIIME1), in line with other studies (Callahan et al., 2017; Nearing et al., 2018). In contrast to Deblur, which uses a static error model to correct raw sequences, DADA2 computes an error model for each sequencing run based on potentially all samples (up to 1 million reads), requiring a re-analysis when only a subset of the initial samples is used in the final reporting. As a consequence, DADA2 requires much more computing time. However, Deblur will miss all amplicons that fall below a required length truncation threshold (e.g., 250 bp in this study) because all shorter amplicons are discarded, thus 1.36% of all sequences in the SILVA v132 database are ignored (Supplementary Figure 5). Furthermore, all amplicons that are longer than the length threshold of 250 bp are cut and therefore essential data is lost. For example, 70% of all sequences in SILVA v132 (99% identity clustered and V4 region extracted) are 253 bp long and are therefore cut by 3 bp, losing >1% of data (Supplementary Figure 5). On the other hand, DADA2 requires choosing read-trimming cutoffs according to data quality, however, there are no defined rules for selecting these cutoffs and, without having a clear expectation of the result, it appears impossible to find the optimal solution. Essentially, operating Deblur seemed riskier than DADA2 because sequences that are below a chosen cutoff can be lost and overlooked using Deblur. Another advantage of DADA2 in our study was the high proportion of recovered sequences and taxa that were specific for each environmental sampling site. Nearing et al. (2018) observed the same trend and suspected that this was due to DADA2’s unique way to create pooled error profiles followed by sample-by-sample ASV picking. This implies that DADA2 might be better in separating similar sequences from different samples than methods that pick sequences from pooled samples (e.g., Deblur, QIIME1, Mothur), however, it is not possible to test this hypothesis with the investigated datasets in the present study.

#### Taxonomic Identification

At genus level, there were substantial differences in the taxonomic overview (presented as bar plots), particularly for the top five most abundant genera, that each method provided. While mock datasets are often analyzed at lower phylogenetic levels, e.g., genus (Almeida et al., 2018; Nearing et al., 2018), environmental datasets are also often shown at higher levels, e.g., phylum (de Voogd et al., 2015; Oliveira et al., 2017). This might be due to the increasing complexity of graphs with increasing microbial diversity. For example, genera below one percent abundance accounted for 75% of the total abundance in the highly diverse soil and sediment samples, investigated in this study, and were better represented by higher taxonomic levels such as phylum, where less than 10% abundance was accounted for when summing up all taxa with less than 1% abundance. However, in lower diversity habitats, i.e., groundwater and river water, the majority of genera were present at above one percent abundance and were reasonably well represented in stacked bar graphs at genus level. Of great concern is the low reproducibility among methods at genus level compared to phylum level. Showing low taxonomy levels down to genus (but not species) was only acceptable when using denoisers, i.e., QIIME2 with DADA2 or Deblur, and should be approached with caution when using OTU picking methods, i.e., Mothur and QIIME1, or taxonomic binning by MEGAN. This is because OTU methods and taxonomic binning performed worse on mock datasets than denoisers, and denoisers reported very similar genera for individual environmental samples. Relative abundance cutoffs were recommended for OTU methods (Bokulich et al., 2013) but these were dependent on the studied samples (i.e., different optimal cutoffs for different methods and mock datasets) and also removed low abundance taxa that might be important (Sogin et al., 2006; Pester et al., 2010; Jousset et al., 2017). The accuracy of the taxonomic representation decreased with decreasing taxonomic ranks and was best for QIIME2 until genus level but was unreliable at species level for all methods. Better taxonomic resolution and classification might be achieved by investigating a larger fraction of the genome such as the full 16S rRNA gene. The V4 sub-region is a good choice because it allows complete overlap of paired-end sequences, thus reducing sequencing errors (Kozich et al., 2013), and it closely resembles the phylogenetic signal of the whole 16S rRNA gene (Yang et al., 2016). The V4 sub-region was therefore also the focus of this study. Compared to sequencing a short region of the 16S rRNA gene with Illumina technology, whole 16S rRNA gene sequencing with Pacific Biosciences (PacBio) technology generates better results in terms of taxonomic resolution (Schloss et al., 2016). PacBio circular consensus sequences (CCS) are produced by reading a circular short sequence (1 to 20 kb), such as the full 16S rRNA gene sequence, several times, thus achieving comparably low error rates similar to Illumina sequencing (Singer et al., 2016). High quality analysis is promised through DADA2 that was recently adapted to be able to denoise PacBio CCS (Callahan et al., 2019). However, PacBio CCS technology is currently not competitive in terms of sequencing depth, price, or availability. Targeting the even longer 16S-ITS-23S sequences of the *rrn* operon with Oxford Nanopore Technologies (ONT) sequencing allowed a high resolution at species level in a recent study (Cuscó et al., 2018). ONT sequencing is continuously improving and, similar to PacBio’s CCS technology, consensus reads are enhancing the accuracy of amplicon sequencing by a large margin, however, ONT’s sequencing accuracy is currently still considered inferior compared to Illumina or PacBio (Calus et al., 2018).

#### Alpha-Diversity

Shannon’s Diversity Index is relatively insensitive to low abundant features (i.e., OTUs, ASVs or taxa) because it uses quantitative information (Shannon, 1948) and the best possible estimates of Shannon’s Diversity Indices were calculated based on QIIME2 using DADA2. The closest resemblance of Faith’s PD required filtering for above 0.01% relative abundance for QIIME2 using Deblur or above 0.1% for QIIME2 using DADA2. Faith’s PD is a qualitative measure (Faith, 1992) and therefore sensitive to the number of features irrespective of their abundance. Qualitative measures are better estimated on high confidence (e.g., high abundant) features, especially for error-prone OTU methods (Bokulich et al., 2013). Taking into account the high number of low-abundance, false-positive sequences in our study, quantitative diversity indices should always perform better on unfiltered data than qualitative measures, a finding that was also earlier reported (Haegeman et al., 2013). An unsuitable approach was to simply count OTUs/ASVs as diversity estimator because this resulted in an overestimation (QIIME1, Mothur, QIIME2 using DADA2) or in an underestimation for low abundant expected taxa (QIIME2 using Deblur).

#### Beta-Diversity

Similar to alpha diversity measures, quantitative beta diversity methods are expected to perform better than qualitative ones, when expecting inaccurate, low abundant features (Lozupone et al., 2007). However, in this study quantitative Bray-Curtis dissimilarity showed a similar sample discrimination as the qualitative Unweighted UniFrac distances. Unweighted UniFrac ignores relative abundances but takes phylogenetic distances into account and, thus, interprets phylogenetically similar sequences between samples as a smaller beta-diversity distance compared to phylogenetically distant sequences.

Beta-diversity distances were relatively similar between analysis methods despite the high variability in taxonomic classification. Bray-Curtis dissimilarity has been also shown to be robust for OTUs or ASVs produced by UPARSE (Glassman and Martiny, 2018). The underlying data structure (i.e., raw sequencing reads, OTU or ASV) for calculating beta-diversity distances is generally similar but mapping sequences to taxonomies performed differently. This is because the methods use very different approaches to resolve taxonomic classification (Almeida et al., 2018). These differences in taxonomic classification are expected to be larger for complex communities with sequences that are poorly represented in databases such as environmental samples and smaller for well-characterized communities such as those stemming from the human gut.

### The nf-core/ampliseq Pipeline Eases 16S rRNA (Gene) Amplicon Analysis Considerably

The pipeline nf-core/ampliseq was implemented following the best analysis method identified here, QIIME2 with DADA2, is independently citable using a Zenodo DOI (Straub and Peltzer, 2019), and can be found in the nf-core collection^5^ (Ewels et al., 2019) to support data analysis that follows FAIR principles (Wilkinson et al., 2016). We opted for nf-core due its strong focus on reproducibility, its strong focus on best practices for scientific software and the unlimited scalability options coming with an nf-core workflow. We argue (in line with the nf-core community) being able to reproduce scientific results is of utmost importance for computational approaches in biosciences. However, it is still notoriously challenging to develop analysis pipelines that are fully reproducible and interoperable across multiple systems and institutions – primarily due to differences in hardware, operating systems and software versions. This is the gap that our pipeline implementation fills for 16S data analysis.

The pipeline nf-core/ampliseq has extensive documentation and excels in reliability, simplicity of usage, reproducibility and efficiency. nf-core/ampliseq will prove valuable because it has minimal software requirements (nextflow, Java, Unix), is easy to use (minimal parameter input: folder containing raw data and primer sequences), and uses computational infrastructure optimally (e.g., hpc job schedulers or cloud computing). All required software dependencies are bundled in containers and are automatically used by this workflow whenever an analysis is performed with a pipeline release. The addition of metadata allows for group comparisons and statistical analysis. Additionally, unwanted sequences can be removed by taxa (default are mitochondria and chloroplast), prevalence or count cutoffs. The output ranges from quality checks (e.g., raw read data, denoising success, alpha-rarefaction) to interactive bar plots, analysis of composition of microbiomes (alpha- and beta-diversity, ordination plots, differential abundant taxa including statistical tests), and tables in text format for further analysis with additional software such as R (R Core Team, 2018).

Currently, nf-core/ampliseq supports solely Illumina-based sequencing analysis but it is planned to allow for (nearly) full length 16S rRNA (gene) amplicon sequencing analysis with PacBio technology using a recent implementation of DADA2 (Callahan et al., 2019). Unfortunately, ONT long reads have currently a too high error rate for ASV tools such as DADA2 but read clustering is an option (Calus et al., 2018) and might be integrated in the future. While any kind of QIIME2 pre-trained database for taxonomic classification is theoretically possible to use with nf-core/ampliseq, only the most updated SILVA database (v132) is currently supported in all detail but more choice is desirable, e.g., UNITE for fungal ITS (Nilsson et al., 2019) is going to be implemented. The aim is to make nf-core/ampliseq the optimal choice not only for Illumina-based 16S rRNA (gene) amplicon sequencing but to expand it to other sequencing methods and to additional phylogenetic or functional gene analysis.

## Data Availability Statement

The datasets presented in this study can be found in online repositories. The names of the repository/repositories and accession number(s) can be found below: https://www.ncbi.nlm.nih.gov/bioproject/PRJNA563986.

## Author Contributions

DS and SK designed the study in discussion with NB, AL-F, and SN. DS analyzed and interpreted the data. DS (major part) and SK wrote the manuscript with the help of SN and NB. NB and AL-F coordinated sampling and processing of environmental material. DS and AP wrote the analysis pipeline nf-core/ampliseq. All authors commented and approved the final manuscript.

## Conflict of Interest

The authors declare that the research was conducted in the absence of any commercial or financial relationships that could be construed as a potential conflict of interest.

## Footnotes

1 https://www.bioinformatics.babraham.ac.uk

2 https://github.com/qiime2/q2-demux

3 https://github.com/qiime2/q2-feature-classifier

4 www.anaconda.com

5 https://nf-co.re

## Supplementary Material

Supplementary Data Sheet 1 Supplementary Text, Tables (1–5,7), and Figures (1–5).

Supplementary Table 6 Five most abundant genera for each sampling site.
