# Supplementary material for: Interpretations of Environmental Microbial Community Studies Are Biased by the Selected 16S rRNA (Gene) Amplicon Sequencing Pipeline
Source: Front Microbiol. 2020 Oct 23;11:550420. doi: 10.3389/fmicb.2020.550420 (PMC7645116; doi:10.3389/fmicb.2020.550420)
Supplement: Supplementary Data Sheet 1 — Supplementary Text, Tables (1–5,7), and Figures (1–5). [file Data_Sheet_1.pdf]

Supplementary Material

1 Supplementary Text

Customized parameters for each pipeline were chosen individually for each dataset, based on standard operating procedures (SOPs), published literature, comparative tests of different parameters, and online resources. Sometimes the published recommendations or guidelines were easy to follow, but this was not always the case.

A required quality setting of DADA2 was the selection of read cutoffs. These read cutoffs needed to be determined for each dataset. The selection of DADA2 cutoffs was informed by the quality of reads, the expected length of the amplicon, and the sequencing read length, as frequently recommended and discussed by the developers of DADA2 and experienced users in several online resources. Unfortunately, there were no definitive rules or numbers to guide the choice of these cutoffs. DADA2 requires for read merging at least 12 bp overlap of forward and reverse reads but 20 bp overlap were recommended. The expected amplicon lengths for the investigated mock datasets were 250 bp-254 bp. The cutoffs for the Balanced dataset were easily determined, because the data showed a dramatic quality drop (median Phred Quality Score) after 200 bp and 120 bp for forward and reverse reads, respectively, and we therefore chose these values. For the Extreme dataset, the read quality dropped much earlier, specifically at 120 bp and 100 bp for forward and reverse reads, respectively. But these values (resulting in 220 bp total read length) would not allow any overlap of forward and reverse reads (at least 274 bp total read length was required). A good compromise between read quality and required total read length seemed 160 bp and 120 bp for forward and reverse reads, respectively. Choosing the read cutoffs for the HMP dataset seemed less obvious than compared to the other datasets, because the quality of the forward reads started to decline already at 120 bp and the quality of the reverse reads was very low from the start. Consequently, keeping a long fragment of the forward read and a relatively short reverse read seemed the optimal choice. Finally, 200 bp and 120 bp were chosen. For environmental samples, more than 95% of expected amplicons were between 251 bp and 256 bp in length (Supplementary Figure 5). With 180 bp read length (for both, forward and reverse reads), we chose relatively high read cutoffs for this dataset because in all three sequencing runs (using the same Illumina MiSeq instrument) the quality of forward and reverse reads was similarly high and started to decrease at around 180 bp.

In order to standardize the selection of DADA2 read length cutoffs, nf-core/ampliseq is attempting to determine the read cutoffs automatically by a median Phred Quality Score cutoff (if no read cutoffs are provided in the command line). However, this solution may fail (a warning reminds the user to check QC files) when the quality cutoff is too low or too high for a specific dataset: DADA2's quality filter removes too many reads when the quality cutoff is set too low (but by allowing more low quality reads to pass the filter, too many reads may contain errors and the method fails) and paired read merging might fail for most amplicons when the quality cutoff is set too high. There were several ideas already to find objective DADA2's read cutoffs, but until now no robust method was implemented in published tools (e.g. to provide information about the expected amplicon length and then iterate through quality cutoffs, to find a compromise between high quality and paired-end read overlap length for merging).

Deblur's sequence length cutoff for the mock datasets was informed by the shortest expected sequence (250 bp or 252 bp, depending on the dataset). For environmental samples, it was chosen based on a trade-off between keeping as many potential ASVs as possible but trimming the lowest number of nucleotides to keep the loss of information at a minimum. Therefore, sequences extracted with primer sequences from the SILVA 132 (99% clustered) database were evaluated for their length (Supplementary Figure 5) and a sequence length cutoff of 250 bp was chosen. This cutoff retained more than 98.6% of all sequences in SILVA and trimmed 1.45% of all bases from longer amplicons. Choosing for example 251 bp would remove additional 0.6% (44% more) of all sequences in SILVA but decreasing it to 249 bp would only add 0.32% (24% more) sequences but trim an additional 0.4% (28% more) nucleotides.

The reference alignment in Mothur was cut to the approximate expected amplicon region (position 11894-25319) and then refined to the majority of aligned reads. Specifically, to the start of the 2.5 percentile and the end of the 97.5 percentile, i.e. for Balanced dataset at position 1968-11546 and for all others at position 1968-11550, and therefore retaining at least 95% of all sequences.

2 Supplementary Figures and Tables

2.1 Supplementary Tables

Supplementary Table 1. Details on the analyzed mock datasets.

	**Balanced**	**HMP**	**Extreme**
Dataset ID	SAMEA3298272	130403 / Mock1	SRR2990088
Direct link	https://www.ncbi.nlm.nih.gov/sra/ERX768310	https://www.mothur.org/MiSeqDevelopmentData/130403.tar	https://www.ncbi.nlm.nih.gov/sra/SRR2990088
Reads	593,868	613,352	2,040,485
Mean quality	34.72	30.50	31.17
Trimmed forward primer	GTGBCAGCMGCCGCGGTAA	GTGYCAGCMGCCGCGGTAA	-
Trimmed reverse primer	GACTACHVGGGTATCTAATCC	GGACTACNVGGGTWTCTAAT	-
Citation	(Schirmer et al., 2015)	(Kozich et al., 2013)	(Callahan et al., 2016)

Supplementary Table 2. Information on environmental samples.

**Sample name**	**Long name**	**Coordinates (Latitude [N], Longitude [E])**	**Sampling date (dd.mm.yyyy)**
GW 1	Groundwater 1	48.5884, 8.8436	24.01.2018
GW 2	Groundwater 2	48.5397, 8.9629	30.01.2018
Soil 1	Soil 1	48.542525, 8.95162	17.09.2018
Soil 2	Soil 2	48.542502, 8.960411	17.09.2018
Sed. 1	Sediment 1	48.5440, 8.9666	25.09.2018
Sed. 2	Sediment 2	48.5395, 8.9636	25.09.2018
RW 1	River water 1	48.5613, 8.9030	28.09.2018
RW 2	River water 2	48.5656, 8.8971	28.09.2018

Supplementary Table 3. Availability of containerized 16S rRNA (gene) amplicon analysis software, that allow access to the exact software environment used.

Software	Singularity pull to container
Trim Galore! v0.4.5	shub://qbicsoftware/qbic-singularity-trimgalore:v0.4.5
Qiime v1.9.1	docker://quay.io/biocontainers/qiime:1.9.1--np112py27_1
QIIME2 q2cli v2018.06	docker://qiime2/core:2018.6
ClipAndMerge v1.7.4	shub://qbicsoftware/qbic-singularity-clipandmerge:v1.7.4
MALT v0.4.0	shub://qbicsoftware/qbic-singularity-malt:v0.4.0
Mothur v1.40.5	docker:// quay.io/biocontainers/mothur:1.40.5--hb16dc7f_0

Supplementary Table 4. Availability of SILVA v132 reference database files for analysis.

Database	Direct link
MALT	https://www.arb-silva.de/fileadmin/silva_databases/release_132/ Exports/SILVA_132_SSURef_Nr99_tax_silva.fasta.gz
QIIME1 & QIIME2	https://www.arb-silva.de/fileadmin/silva_databases/qiime/Silva_132_release.zip
Mothur	https://www.mothur.org/w/images/3/32/Silva.nr_v132.tgz

Supplementary Table 5. Recovered sequences for three mock datasets (Balanced, HMP and Extreme) analyzed with three pipelines (QIIME1, QIIME2 and Mothur) with different settings (QIIME1 and Mothur: 99 and 97% similarity for clustering into OTUs; QIIME2: DADA2 or Deblur for ASV detection) compared to reference sequences.

Mock dataset	Mismatches	Mothur		QIIME1		QIIME2	
		99[a]	97[a]	99[a]	97[a]	DADA2[b]	Deblur[b]
Balanced (62 seq.)	0	47	48	58	58	59	56
	1	21	2	727	721	0	0
	>1	1,613	477	689	223	30	18
	F-score	0.05	0.16	0.08	0.11	0.78	0.82
HMP (25 seq.)	0	17	20	21	21	22	20
	1	22	2	1,918	1,895	1	0
	>1	1,517	524	2,237	910	50	15
	F-score	0.02	0.07	0.01	0.01	0.45	0.67
Extreme (35 seq.)	0	22	21	29	29	25	15
	1	32	2	803	807	1	0
	>1	7,955	344	4,713	575	24	3
	F-score	0.01	0.10	0.01	0.04	0.59	0.57

seq.: expected unique amplicon sequences

[a]similarity (%) at which sequences are clustered into operational taxonomic units (OTUs)

[b]ASV calling software

Supplementary Table 7. Stress values for NMDS, plotted in Figure 9 (Comparison of beta-diversity plots for environmental samples). All stress values are below 0.06, indicating a fair (<0.1) or even good fit (<0.05).

Pipeline	Bray-Curtis	Unweigthed UniFrac
MEGAN	0.036	n.d.
Mothur 99	0.044	0.046
Mothur 97	0.040	0.020
QIIME1 99	0.042	0.052
QIIME1 97	0.040	0.022
QIIME2 DADA2	0.055	0.037
QIIME2 Deblur	0.055	0.027

2.2 Supplementary Figures

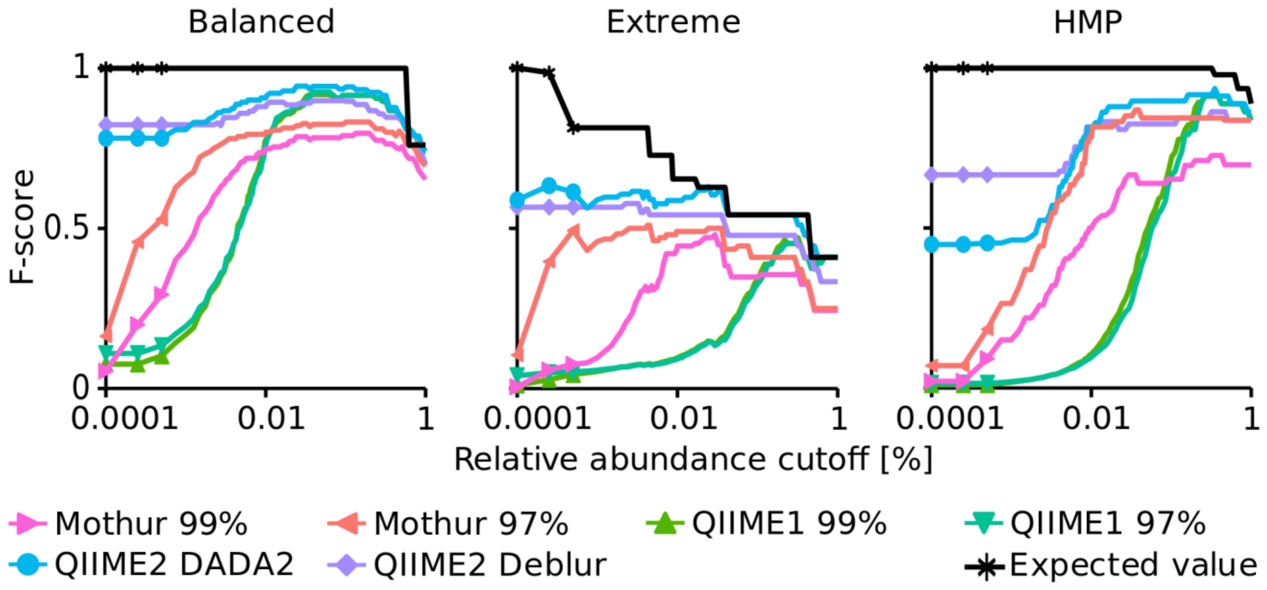

Supplementary Figure 1. F-score of sequence (OTU/ASV) recovery dependent on a relative abundance cutoff (up to 1%).

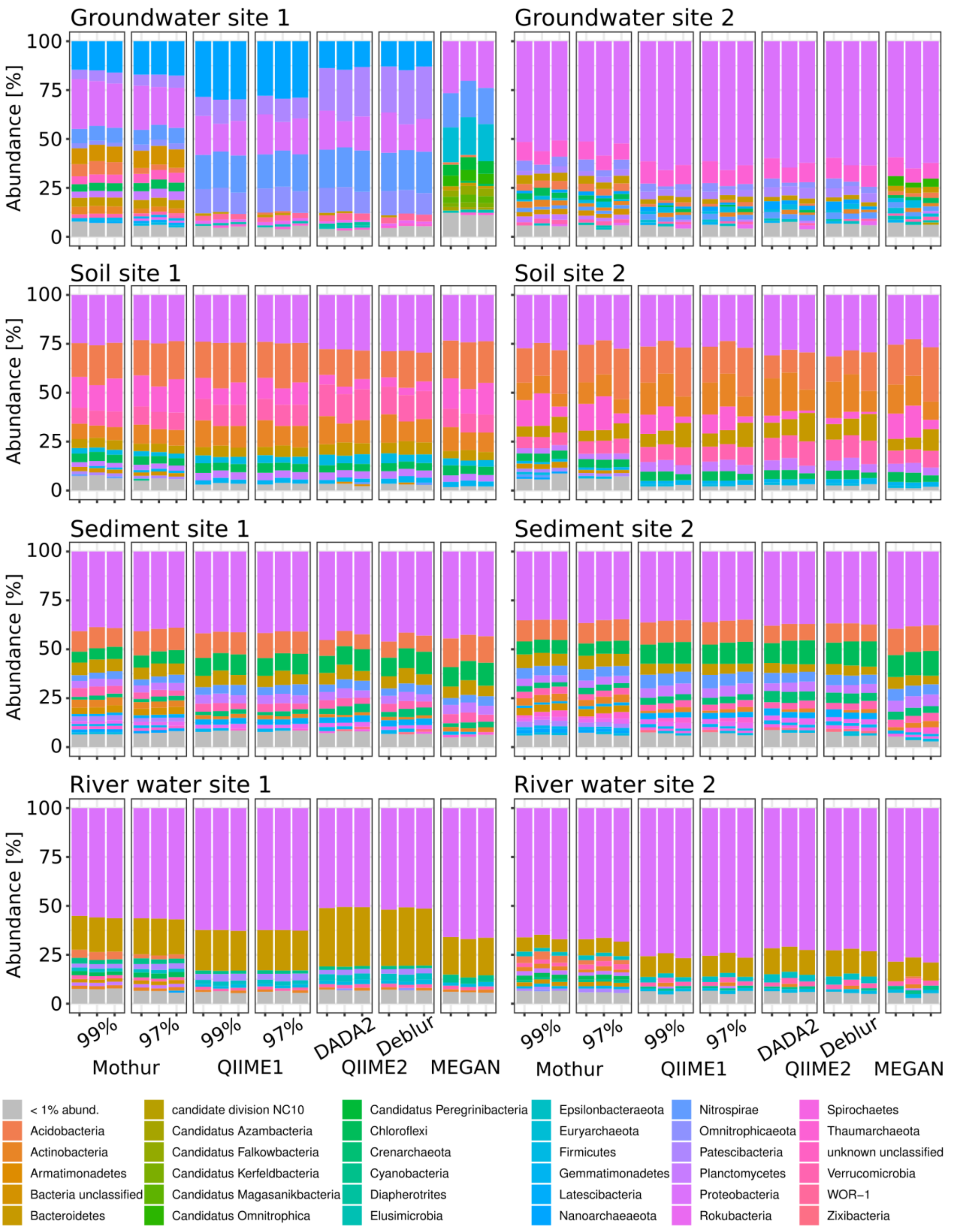

Supplementary Figure 2. Relative abundance of microbial taxa at phylum level for all habitats, sampling sites, sample replicates and analysis methods. Phyla are shown in the same colors throughout the figure. Phyla <1% relative abundance are shown in grey color.

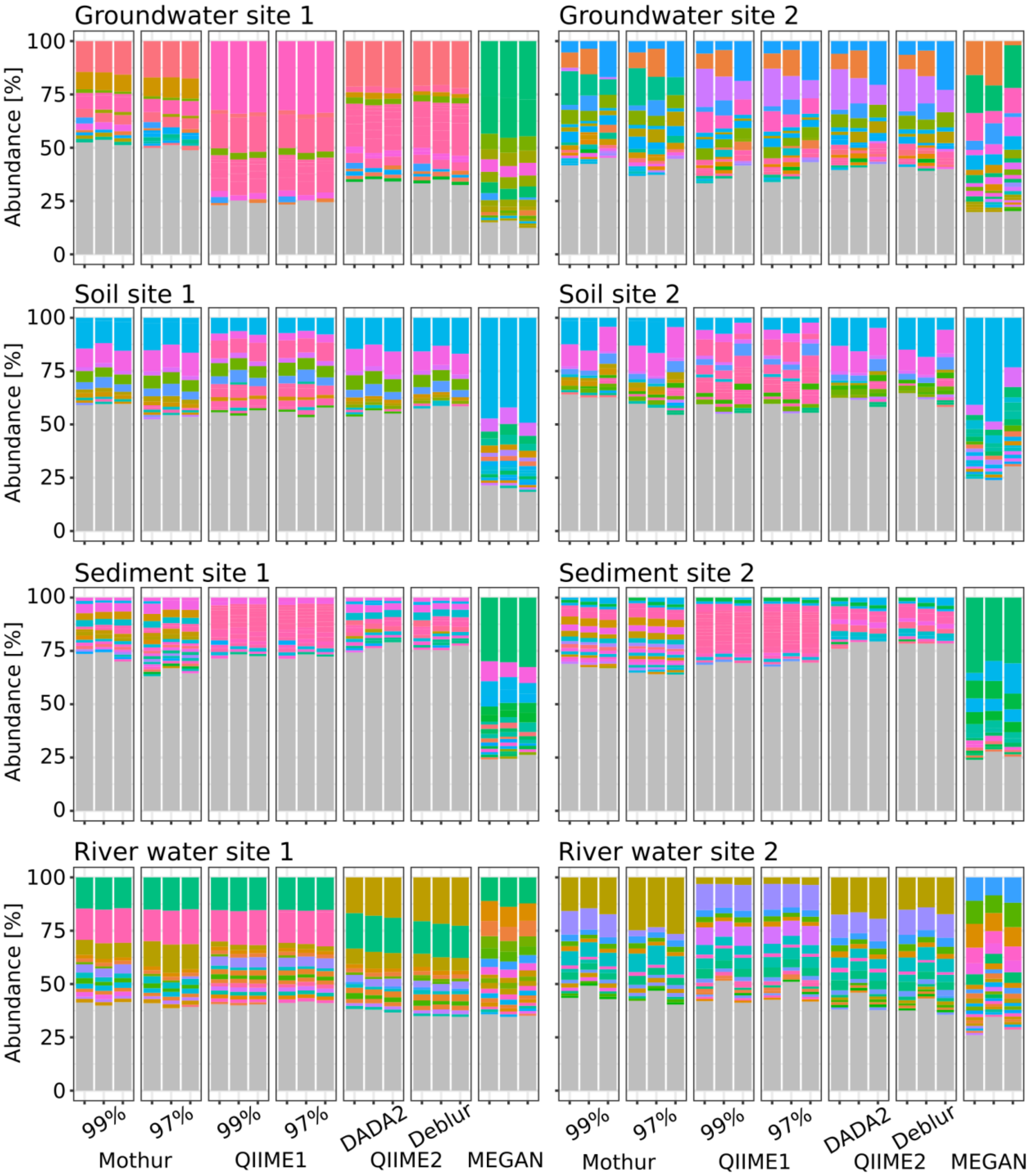

Supplementary Figure 3. Relative abundance of microbial taxa at genus level for all habitats, sampling sites, sample replicates and analysis methods. Genera are shown in the same colors throughout the figure. Genera <1% relative abundance are shown in grey color. The aim of this figure is to give an overview about similarities between methods but the color code of the 166 genera is too similar to distinguish them reliably, therefore the color legend is not shown.

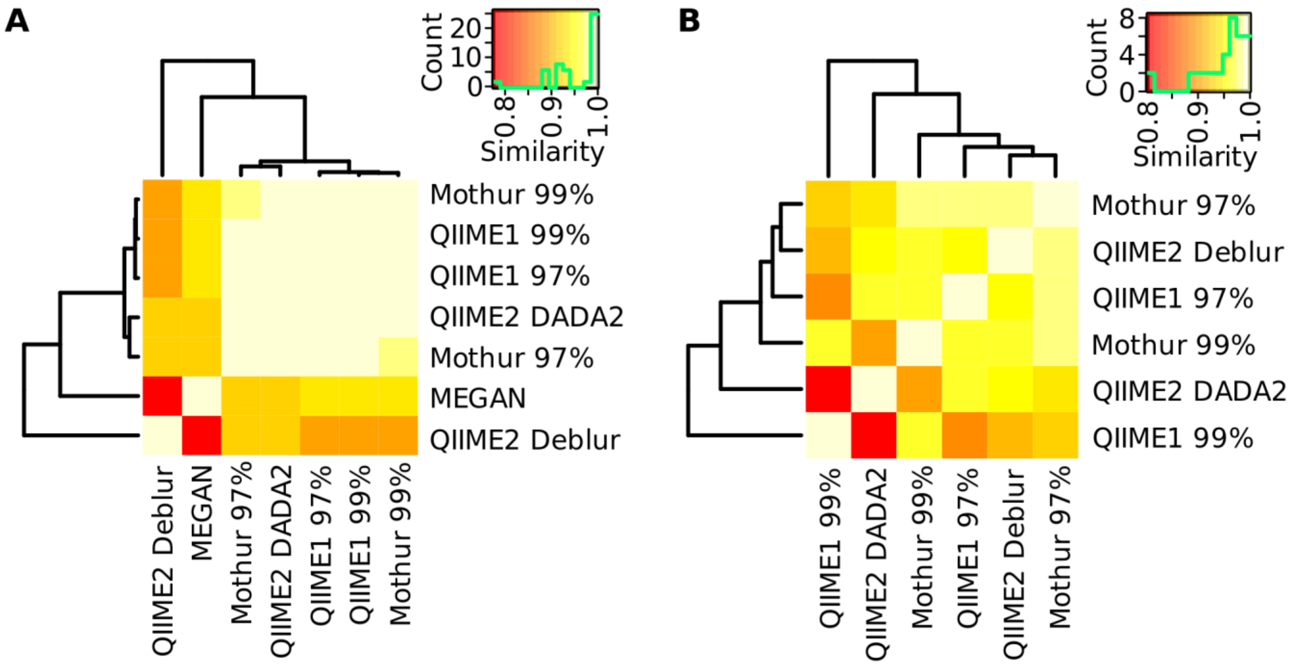

Supplementary Figure 4. Procrustes Similarity Indices between (A) Bray-Curtis dissimilarity or (B) Unweighted UniFrac distance NMDS plots in Figure 9. Procrustes Similarity Index is equal to zero when NMDS coordinates are uncorrelated and equals one if plots are identical when being rotated or scaled.

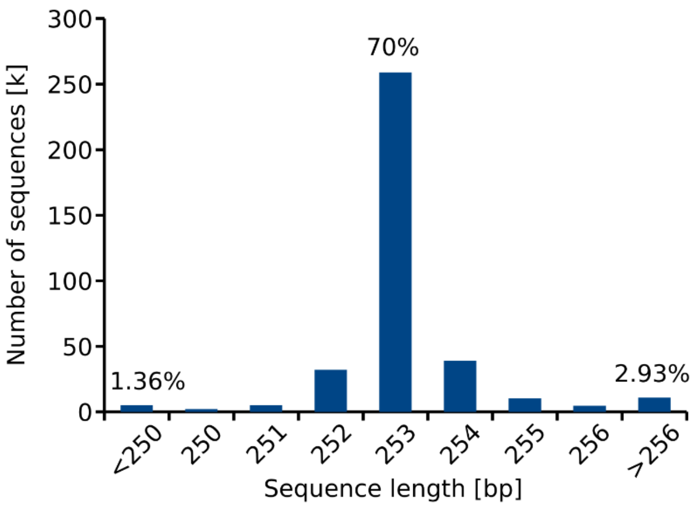

Supplementary Figure 5. Length distribution of the V4 region using primers 515f and 806r extracted from the SILVA v132 database (clustered at 99% similarity) with QIIME2. The majority of sequences (70%) is 253 bp long, sequences from 251 bp to 256 bp sum up to more than 95%. However, 1.35% or 2.93% of all sequences are below 250 bp or above 256 bp long, respectively.

3 References

Callahan, B.J., McMurdie, P.J., Rosen, M.J., Han, A.W., Johnson, A.J.A., and Holmes, S.P. (2016). DADA2: High resolution sample inference from Illumina amplicon data. *Nature methods* 13(7), 581-583. doi: 10.1038/nmeth.3869.

Kozich, J.J., Westcott, S.L., Baxter, N.T., Highlander, S.K., and Schloss, P.D. (2013). Development of a Dual-Index Sequencing Strategy and Curation Pipeline for Analyzing Amplicon Sequence Data on the MiSeq Illumina Sequencing Platform. *Applied and Environmental Microbiology* 79(17), 5112-5120. doi: 10.1128/AEM.01043-13.

Schirmer, M., Ijaz, U.Z., D'Amore, R., Hall, N., Sloan, W.T., and Quince, C. (2015). Insight into biases and sequencing errors for amplicon sequencing with the Illumina MiSeq platform. *Nucleic Acids Research* 43(6), e37-e37. doi: 10.1093/nar/gku1341.
